# Interleukin-13 peptide vaccine induces protective humoral immunity in murine asthma models

**DOI:** 10.18632/oncotarget.19950

**Published:** 2017-08-04

**Authors:** Fengbo Wu, Yan Huang, Peng Zhang, Chunting Wang, Yaomei Tian, Lian Lu, Gu He, Li Yang

**Affiliations:** ^1^ Department of Pharmacy, State Key Laboratory of Biotherapy, West China Hospital, Sichuan University, Collaborative Innovation Center of Biotherapy, Chengdu, Sichuan, P.R. China; ^2^ Department of Radiation Oncology, Sichuan Cancer Hospital & Institute, Sichuan Cancer Center, School of Medicine, University of Electronic Science and Technology of China, Chengdu, Sichuan, P.R. China

**Keywords:** interleukin-13, asthma, synthetic peptide, vaccine, Pathology Section

## Abstract

This study presents a rational design approach to discovery synthetic peptide vaccine candidates from endogenous proteins for chronic non-infectious diseases immunological therapeutics. The approach described the screening of key antigenic amino acid residues of the interleukine-13, which is up-regulated expression in asthma, followed by the development of immunological helper epitope peptides *via* an integrative computational and experimental method. Notably, this totally synthetic peptide vaccine was capable of stimulating humoral immune responses much stronger than those of parental antigenic peptides by enhancing the efficiency of antigen presentation, and had effective treatment in mouse asthma models. Our approach offers new possibilities to discovery therapeutic peptide vaccine candidates for chronic non-infectious diseases, with highly consolidated in silico and animal disease models for fast iterative screening.

## INTRODUCTION

Asthma is an atopic disorder with features that include airway inflammation and airway hyper-responsiveness (AHR). [1] Dysfunctional regulation of interleukins (IL) often associates with different pathogenesis processes of asthma. [2–4] Nowadays, the administrations of soluble receptors or monoclonal antibodies (mAb) of interleukins are efficient treatment approaches for asthma and other allergic disorders. [5] However, these therapeutic approaches possess some shortcomings, e.g. limited half-life, frequent and inconvenient injections, relatively large dosage and costly treatment charge. Most recently, mepolizumab (IL-5 mAb) has been approved by FDA (Food and Drug Administration) for the treatment of adults with severe asthma; however, its high therapeutic costs brought the manufacturer GSK was condemned by the public immediately. Therefore, novel strategies to overcome these disadvantages are highly needed.

IL-13 is characterized as a prime effector in asthma pathology, and is found to be necessary and sufficient for all the features of allergic asthma responses. Up to now there are over 500 registered clinical studies of peptide vaccines for preventive or therapeutic purpose in different disease conditions, and 11 studies among them have progressed to phase III level of development until middle 2015. Based on IL-13 is to be one of the most important modulators in asthma pathology, vaccines, monoclonal antibodies and soluble receptors to block IL-13 have been developed, which are mainly keen on developing recombinant protein antagonists to block IL-13 signal of target cells or neutralize the functional of extracellular IL-13. [6, 7] However, these approaches also presented many disadvantages, such as high-dose requirements, the high expenses of facilities, the stability of the antagonists, et al.

In the previously researches, we and other groups had reported effective asthma vaccines on the basis of IL-13. [8–10] Because these IL-13 antigenic epitopes were only weakly immunogenicity and/or immune-tolerance cannot stimulate strong immune responses, to enhance their immunogenicity, antigenic epitopes have been covalently conjugated with immune activators or carriers, such as keyhole limpet hemocyanin (KLH) or virus-like particle (VLP). However, most of the carriers are too highly immunogenic to suppress the production of specific anti-interleukin-13 antibody. To induce a specific and potent immune response, synthetic peptide vaccine candidates have shown their advantages, because only the epitopes required for desirable T cell and/or B cell mediated immune responses are incorporated into the constructs. [11–13] Nevertheless, there are just a limited number of conjugated IL-13 epitopes vaccines capable of inducing effective immune responses, and none synthetic IL-13 peptide vaccine is reported before. This has created the demands for IL-13 peptide vaccines that can stimulate specific antibody production, and prevent airway inflammation, AHR and epithelial cell proliferation with goblet cell hyperplasia in an acute asthma model.

**Figure 1:**
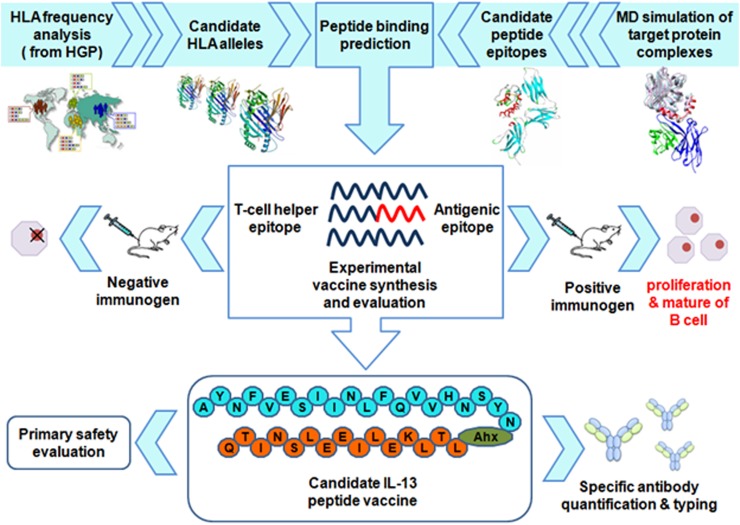
The flowchart for discovery of IL-13 peptide vaccine The figure was drawn by using Adobe Photoshop CS6 (ver: 13.0, Adobe Systems Software Ireland Ltd, CA, USA. http://www.adobe.com)

To construct a peptide vaccine comprised from a IL-13 antigenic epitope and a immune stimulating peptide that are required to induce efficient immune response, distinctive IL-13 epitope candidates and T helper epitope peptides have been screened and conjugated with each other. After lysosomal processing, the IL-13 peptide vaccine candidates are presented on the major histocompatibility complex II (MHC II) in antigen-presenting cells (APCs), thereby resulting in the activation of the helper T cells, which then stimulate the recognized B cell to Produce specific antibodies. In brief, the crystallographic analysis of IL-13 complexes with IL-13/IL-4 receptor or IL-13 mAb (Lebrikizumab), [14] combined with molecular dynamics (MD) simulations, provides new approaches for structure-based immunogenic epitope selection. Based on these specific recognizable IL-13 epitopes identified by MD simulation, the next rational consideration of these peptide vaccines is to improve population coverage through T helper epitope discovery: (i) epitope prediction: given a set of IL-13 antigenic epitopes, candidate T helper epitopes are identified with respect to a set of target HLA alleles which highly expression in human (from human genome project (HGP) reports); (ii) epitope selection: the suitable subset of T helper epitopes is screened from the set of candidate epitopes by knowledge-based methods; and (iii) vaccine synthesis and validation: the peptide vaccines are synthesized from the selected antigenic and T helper epitopes. Moreover, their antigenicity and protective effect are validated in murine asthma models (see Figure 1).

## RESULTS AND DISCUSSION

**Figure 2:**
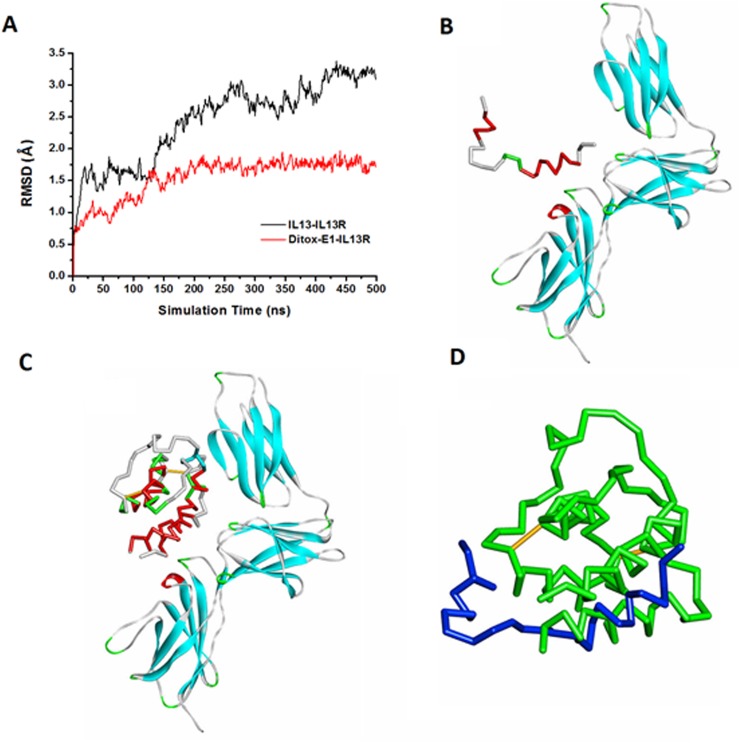
The molecular dynamics simulation of Ditox-E1(V1) and IL-13 to IL-13 receptor **A.** The RMSD of backbone atoms in Ditox-E1-IL13R and IL-13-IL13R complexes; **B.** The equilibrium conformation of Ditox-E1-IL13R; **C.** The equilibrium conformation of IL-13-IL13R; **D.** The superposed equilibrium conformations of IL-13 (green) and Ditox-E1 (blue).

The MD simulations of candidate IL-13 peptide vaccine-IL-13 receptor complexes (using epitope 1 as a representative example, showed in Figure 2) were successfully run for 500 ns. The RMSD (Root Mean Square Derivation) values of the IL-13R and IL-13 antigenic epitopes backbone atoms relative to its initial structures were calculated and plotted to evaluate the stability of the MD trajectories, the RMSD values of the Ditox-E1, Ditox-E2 and Ditox-E3-IL-13R complexes were 0.17, 0.19 and 0.23 nm, respectively. The Supplementary Figure 1 showed the representative IL-13 peptide vaccine, Ditox-E1(V1), the RMSD curve and simulated conformations compared to the IL-13 protein. These results suggested that the MD trajectories of the three complexes were stable, and the conformations of epitopes were maintained so it was reasonable to do the experimental validation. Furthermore, in consideration of the experiments were performed on the Balb/c mice, the candidate epitopes E1, E2 and E3 were transformed to corresponding murine sequences (Supplementary Figure 1).

**Figure 3:**
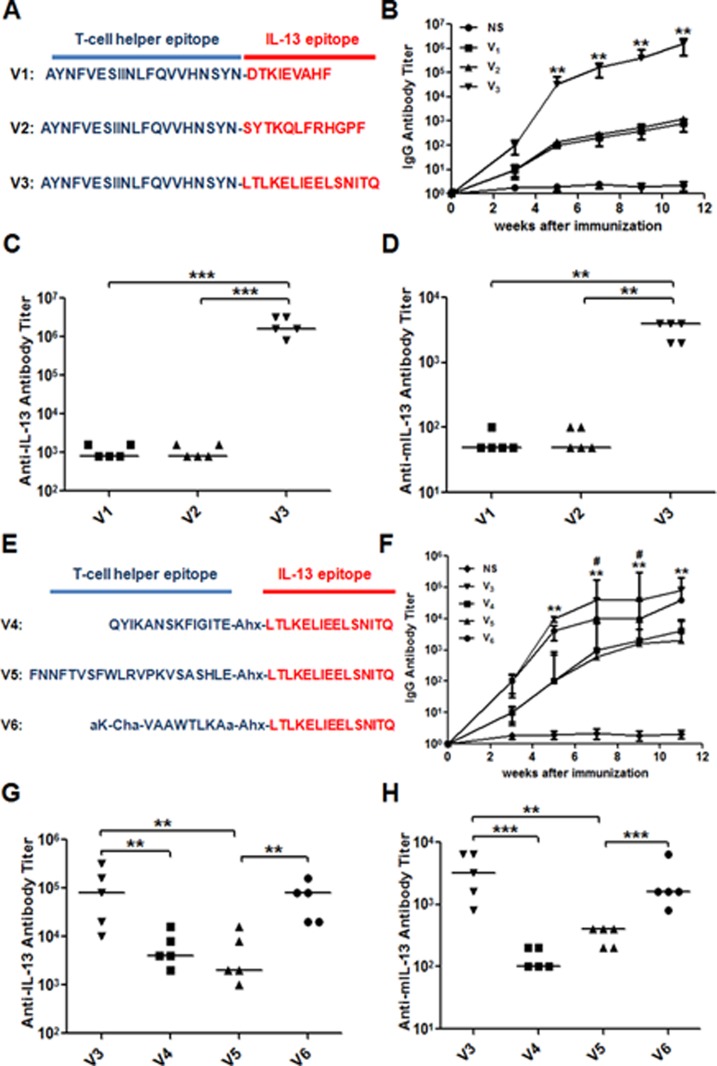
The structure of IL-13 peptide vaccines and specific antibody titer **A.**&**E.** Structures of V1 to V6; **B.**&**F.** average anti-IL-13 antigenic epitope IgG titers changes after immunization; **C.**&**G.** Anti-IL-13 antigenic epitope IgG titers; **D.**&**H.** Anti-IL-13 recombined protein IgG titers.

**Figure 4:**
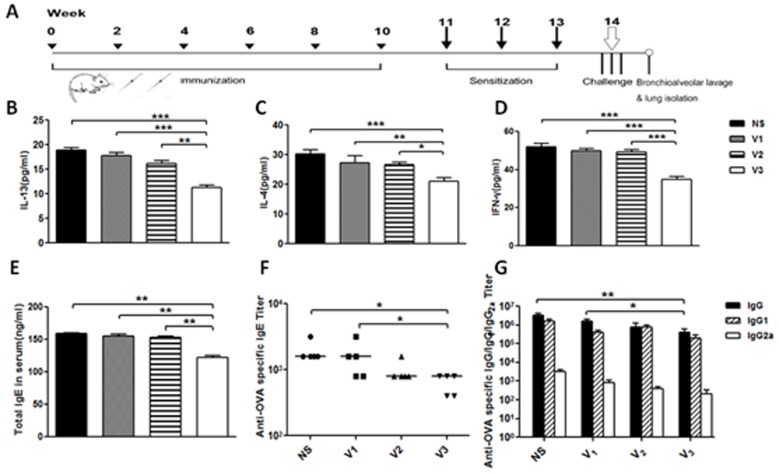
Preliminary evaluation of new asthma vaccines based on diphtheria T helper epitope peptide was measured **A**. Protocols of immunization, sensitization and challenge. **B**. Levels of IL-13; **C**. IL-4; **D**. IFN-γ in BALF, **E**. Total IgE in serum were measured by ELISA kits; **F**.-**G**. OVA-specific antibodies were taken by ELISA; data was reported as mean±SEM. Significant differences were shown as *P < 0.05, **P < 0.01 and ***P < 0.001.

Herein, we firstly synthesized and evaluated several vaccine candidates V1, V2 and V3, which contained different IL-13 antigenic epitopes (E1, E2 and E3, respectively) conjugated to a T helper epitope derived from diphtheria toxin (Ditox382-401, AYNFVESIINLFQVVHNSYN) (Figure 3A). The Ditox382-401 could serve as an adjuvant and induce strong antibody responses in murine models with a commonly employed alum adjuvant. To evaluate the immune response of the antigenic epitopes, V1, V2 and V3 were incubated with alum adjuvant and then injected subcutaneous. All vaccines were administered on weeks 0, 2, 4, 6, 8 and 10. The sera were collected on weeks 3, 5, 7, 9 and 11, the antibody titers were measured by ELISA (enzyme-linked immunosorbent assay) method (Figure 4A). The results showed that V3 elicited the highest immune response and the highest IgG titer against peptide epitope up to 10^6^, and the IgG titers of V1 and V2 were about 10^3^ (Figure 3B). Meanwhile, the IgG titers against recombined murine IL-13 proteins of V1, V2 and V3 were about 5000, 800 and 700, respectively. Compared to E1 and E2 were mainly interacted with IL-13R, E3 was interacted with both IL-13R and IL-4R, previously reported conjugated IL-13 vaccine also suggested E3 to be an efficient immunogenic epitope. According to previous articles, antibody titer just was one of index to evaluate the effective of allergy vaccine. Bronchoalveolar lavage fluid (BALF) and cytokines analysis, together with histological results in asthma model were also very important. Although the asthma pathogenesis is very complex, Th2 cells are considered as a key role in the response of asthma. The results showed that asthma important cytokines, IL-13 and IL-4 which were released by Th2 cells, were obviously downregulated in V3 group. As with cytokine levels of IFN-gamma in BALF, total IgE in serum and antibodies of OVA-specific IgE/IgG were distinctly declined in V3 (Figure 3).Thus, we suggested that E3 may stimulate immunogenic responses, downregulate cytokines in BALF or serum, suppress inflammation/AHR and, consequently, E3 was selected as IL-13 antigenic epitope for further evaluation of T helper epitopes.

**Figure 5:**
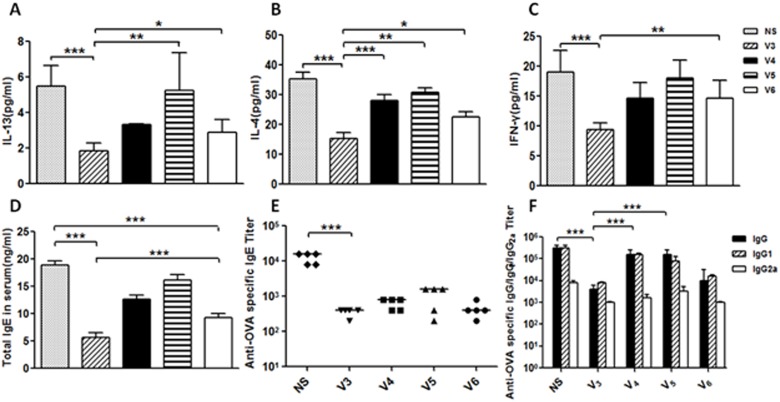
Preliminary evaluation of new asthma vaccines based on E3 epitope peptide was measured **A.**-**C.** After the last immunization of vaccines, IL-13, IL-4 and IFN-γ were tested by enzyme-linked immunosorent assay (ELISA); **D.**Total IgE in serum measured by ELISA; **E.** OVA-specific antibodies were taken by ELISA; **F.** The type-specify of IgG antibody against OVA; data was reported as mean±SEM. Significant differences were shown as *P < 0.05, **P < 0.01 and ***P < 0.001,#*P* < 0.05.

Based on E3 epitope, then we synthesized vaccine candidates V4, V5 and V6, which containing E3 conjugated to other three T helper epitopes derived from tetanus toxin (TT830-843, QYIKANSKFIGITE; TT947-969, FNNFTVSFWLRVPKVSASHLE) and non-natural T helper epitope (PADRE, aK-Cha-VAaWTLKAa). In order to confirm the best T helper epitope peptide, mice were treated as previous protocols with vaccines (V3-V6). Compared with four groups, V3 generated long-lasting and the highest IgG titer against E3 epitope up to 105 at the end of experiment (Figure 3D), IL-13-specific IgG only showed IgG titer against peptide, more ever, to evaluate new vaccine association with natural IL-13 *in vivo*, microplates coating with murine IL-13 protein by ELISA were taken out. V3 in both tests respectively showed highest titer than other three groups (Figure 3D). Other more, cytokine levels of IL-13, IL-4, IFN-gamma in BALF, total IgE in sera and OVA-specific antibodies were significantly decreased (Figure 4). In order to explore whether it has effect on other tissues or not, the weight of mouse were measured during and at the endpoint the experiment. Tissues of heart, liver, spleen and kidney were measured by Hematoxylin-and-eosin staining used an Olympus light microscope equipped with a camera. Combined with the two check points, there were no differences between V3 and other two groups (Figure 5). Thus, V3 vaccine may preliminary serve as an alternative innovative and effective treatment against asthma.

IL-13 and total IgE were the most points cytokines in asthma, to detect the changes of the two cytokines could show whether the mice down-regulated the degree of asthma after receiving candidates vaccines. As studies before, asthma was Th2 type response. The detection of IL-4 and IFN-γ which were the marks in Th2 type could reveal the reactions in immunized mouse. All cytokines were obviously down-regulated by vaccines. Most importantly, IL-13, IL-4 and IFN-γ from immunized mice were significant decreased than those from mice exposed to OVA (ovalbumin) (*P* < 0.001). Besides, such a decrease was generated in level of total IgE from immunized mice (*P* < 0.01). Therefore, the mice received V3 vaccination, the degree of cytokines in asthma was not only reduced, but also the Th2 type response.

OVA can induce asthma in mice model, the level of OVA-specific antibodies is another index to show the changes between groups. OVA-specific IgE level and of V3 group was an obvious mark in asthma model. The OVA-specific IgE was down-regulated than the mice which were injected V1 or V2 vaccine. Furthermore, OVA-specific IgG titer was remarkable down-regulate after immunization with V3 vaccine. In all, V3 vaccine could significantly reduce the allergic in asthma mice.

IL-13 and total IgE were the most points cytokines in asthma. As studies before, asthma was Th2 type response. Except to analyze the changes of IL-13, total IgE in serum, the levels of IL-4 and IFN-γ which belonged to Th2 type response could reveal the reaction in immunized asthma mice. When compared to model mice exposed to OVA, all cytokines in BALF and serum were apparently decreased from V3 vaccination (*P* < 0.001). In general, the vaccination of V3 vaccine produced apparent effects both on cytokine levels and Th2 responses.

After OVA challenge 72 hours, circulating IgE antibody levels were detected to determine whether vaccine V3 could generate a response of OVA-specific T-helper 2 cell. Sera were analyzed by ELISA. The OVA-specific IgE levels in V3 vaccination were apparently decreased than those showed in the other groups to the model group (*P* < 0.001). Otherwise, such a reduction of OVA-specific IgE levels was produced by V3 vaccination to the model group than other groups (*P* < 0.001). It revealed that the V3 vaccine generate a obviously effect in down-regulation of OVA-specific antibody levels.

**Figure 6:**
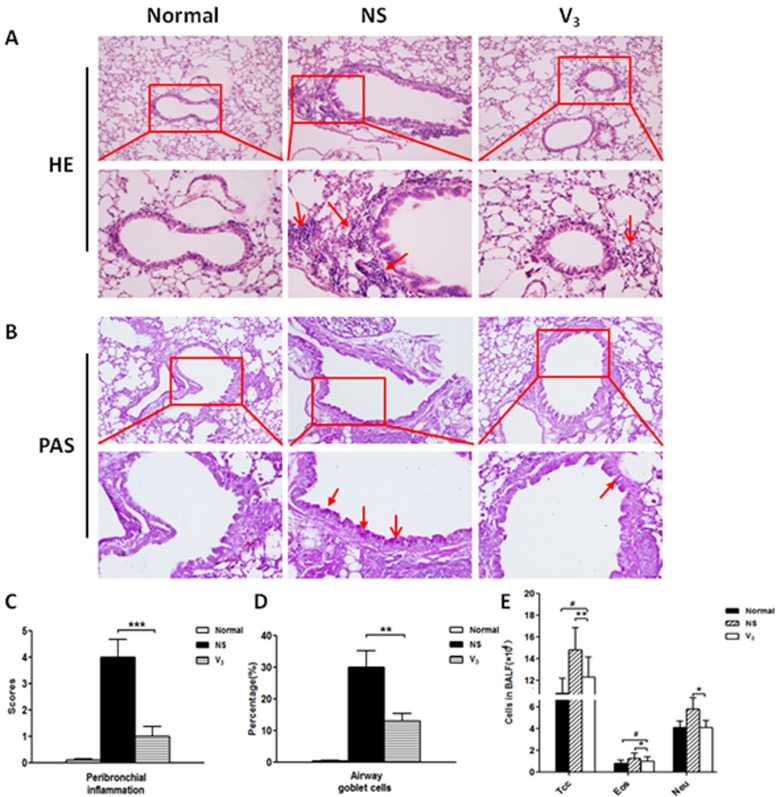
**A**. H&E staining of bronchoalveolar tissues after immunization and OVA sensitization; **B**. PAS staining of bronchoalveolar tissues after immunization and OVA sensitization; **C**.-**D**. Bronchoalveolar inflammation and goblet cells counting in the BALF; **E**. Total cells, eosinophil and neutrophil cells counting in the BALF.

To evaluate airway goblet cell hyperplasia and the accumulation of inflammatory cells around peribronchial after sacrificed mice, lung tissues were collected in formalin-fixed. H&E staining of lung tissues was verified that inflammatory cells around peribronchial and perivascular were suppressed in mice that received V3 vaccine. In contrast, model groups that received saline appeared significant accumulation of inflammatory cells around peribronchial and perivascular compare with V3 group that received vaccine (Figure 6A and 6B). With lung tissue immune cell infiltration, representative images and semi-quantitative scoring analyses of PAS-stained correlated well. In model mice, OVA challenges increased remarkable goblet cells. On the contrary, V3 group showed fewer goblet cells (Figure 6C and 6D). At the end point of experiment, differentiation cell counts in bronchoalveolar lavage fluid (BALF) were performed. V3 vaccine immunization significantly suppressed accumulation of total cells, eosinophil and neutrophil rather than model group (Figure 3E).

**Figure 7:**
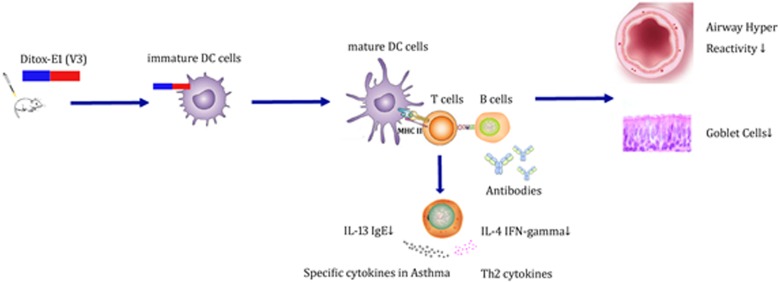
Potential molecular mechanism of IL-13 peptide vaccine in the murine acute asthma model

In the classical antigen presentation pathways, the proteasome process endogenous antigens which were loaded on MHC I molecules in the ER to activate cytotoxic CD8+ T cells. [15–18] More ever, phagocytosis and endocytosis take up exogenous antigens which are processed by lysosome, loaded on MHC II in lysosome-derived organelles for CD4+ T cell activation. To define which of the two pathways activated in CD8+ or CD4+ T cell vaccination, we cultivated dendritic cells, labeled E1 and DiTOX-E1 peptides by The Alexa Fluor 488 Microscale Protein Labeling Kit, treated mature DCs with Lyso-Tracker Red added labeling or anti-LMPS-cy3 added labeling peptides for 1 hour. Confocal microscopic analysis showed that lysosome, treated with Lyso-Tracker Red, was found fused with green labeled peptides. In contrast, proteasome was colocalized with green labeled peptides (See supporting materials). These data demonstrate that while there are both antigen presentation pathways in immune response, E1 and DiTOX-E1 uniquely used the lysosome to cross-presenting MHC II to activate CD4+ T cell responses which could enhanced production of B cells to generate IgG antibodies and T cells to suppress the secretion of cytokines in mouse asthma model (Figure 7).

**Figure 8:**
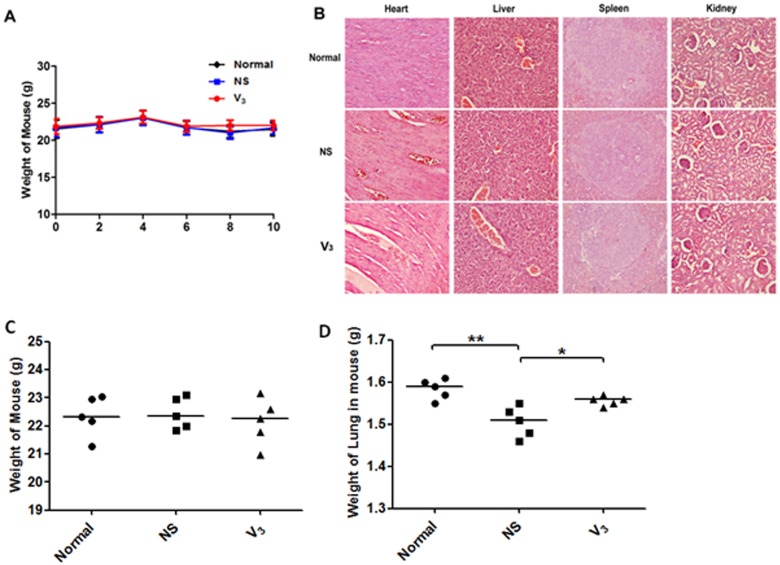
Safe evaluation of V3 vaccine based on diphtheria T helper epitope peptide was measured **A.** At each point of immunization, weight of mouse was taken. **B.** Representative photomicrographs are shown. **C.**-**D.** Weight of mouse and lung in mouse were measured at the end point of the experiment; data was reported as mean±SEM. Significant differences were shown as *P < 0.05, **P < 0.01 and ***P < 0.001.

To primary evaluate the potential toxicity of peptide vaccines, the body weight of all mice were measured every week, the results demonstrated that body weight of all mice remained stable during vaccination (Figure 8A). At the end point of experiment, tissues of heart, liver, spleen and kidney were measured by H&E staining. By using an Olympus light microscope equipped with a camera, compared to normal group, tissues in both model group and V3 vaccination group appeared no apparently changes (Figure 8B). Moreover, the total lung weight of mice in each groups were not changed obviously. In addition, compared to model group, V3 treated group showed fewer lung weight loss, implied the protective effect of V3 in the acute asthma models (Figure 8C and 8D).

During the last decade, peptide vaccine of cancer has represented a hot area of anti-cancer therapeutic investigation world-wide. We have learned how tumor cells escaped from the attack by the immune system in any type of immune-biotherapy. [19–27] Nevertheless, asthma is an abnormal immune status, which is positively modulating the ongoing aberrant immune response. Therefore, it is likely that a therapeutic vaccine should suppress the ongoing autoimmune response, which is quite different from cancer therapeutic vaccine. In addition, interleukin families are significantly effects the progression of asthma, many studies about antibodies and RNA interference targeting the IL-4, IL-5 and IL-13. IL-13 mAbs have entered phase II or phase III clinical trial stages. Although the mAb has some advantages, such as high target selectivity, rapid pharmacodynamics profile and good safety, however, it still has some defects, e.g. expensive therapeutic costs, unexpected reactions with unrelated antigens and potential allergic hazard, which make the discovery of therapeutic vaccine on demanded. In consideration of the peptide vaccine can also inducing specific and continuous antibody-mediated immunoreactions, associated with the relatively low expense and easily to vaccination, we think peptide vaccine targeted to IL-13 might be an attractive therapeutic strategy.

With the rapid progress of structural biology, more and more atomic structures of therapeutic targets have been determined in recent years. Although the drug discovery and molecular biological research benefit from these progresses, the structure-based vaccine discovery has just starting out. [28–34] Our strategy focused on the integrative in silico and experimental screen methods to discovery therapeutic peptide vaccine candidates for chronic non-infectious diseases. The totally synthetic IL-13 peptide vaccine provides a new vision to vaccine discovery, with a simple and well-define structure that should deserve further studies. Our approach offers a fast and convenient to discovery therapeutic peptide vaccine candidates from target protein structures, with highly consolidated in silico and animal disease models for rapid iterative screening.

## MATERIALS AND METHODS

### Selection of high frequency HLA alleles

In general, the distribution of Top20 HLA-DR alleles in Chinese population was selected from The Allele Frequency Net Database (AFND, http://www.allelefrequencies.net/). The screened alleles and their coverage in Chinese population were also listed in Supplementary Table 1, the total coverage from 13.5% (DRB1*09:01) to 1.0% (DRB1*14:05) and total covered 95.9% of the Chinese population. In the Immune Epitope Database (IEDB, http://www.iedb.org), there were six methods for MHC binding were performed to predict the binding affinity between IL-13 epitopes and HLA DR types for the prediction of candidate IL-13 epitopes. The methods including Consensus method, Combinatorial library, NN-align (netMHCII-2.2), SMM-align (netMHCII-1.1), Sturniolo, and NetMHCIIpan.

### Prediction, molecular docking and MD simulation of candidate epitope to HLA alleles

To be a candidate epitope, it ought to bind with MHC with a highly affinity. We selected epitopes from consensus score using six prediction methods from IEDB with a dataset of 20 Chinese HLA-DRB1 alleles. The epitopes bind to MHC class I molecules that were removed first, and then the predicted HLA-DRB1 allele affinity was considered, the top 95 epitopes with 5% or below percentile (Supplementary Table 2) were docked into the IL13 receptor to determine their interaction with IL13R by using the Accelrys Discovery Studio software. Next, the candidate epitopes with higher predicted binding affinity with both HLA-DR alleles and IL13R were shifted to the MD simulation to further confirm their conformation stability between in the IL-13 protein and in the peptide vaccine. It is hard to experimentally determine the interaction between the candidate IL-13 peptide vaccine and IL-13 receptor at the atomic level. Consequently, we have constructed these structures with the aid of a molecular mechanics (MM) method and a molecular dynamics (MD) approach. Initially, the co-complex structure of IL-13/IL-13R was retrieved and downloaded from the PDB database (PDB No. 5E4E), and the structure of candidate IL-13 peptide vaccine were built by employing the Accelrys Discovery Studio software and the Sander module of the Amber 12 package. Prior to MD simulations, each simulation system was subject to energy minimization to avoid steric clashes between the complex and the solvent. In the equilibrium simulations, PME (Particle Mesh Ewald) method was performed to deal with the long-range electrostatic interactions. The cutoff distances for the long-range electrostatic interaction was set to 10 Å, as well as the VDW (van der Waals) interaction. The simulated systems were gradually heated from 0 to 300 K within 100 ps in the NVT ensemble. Finally, the long-time production MD simulations (500 ns) were carried out in an NPT environment under periodic boundary conditions. The SHAKE method was applied to constrain all covalent bonds with a tolerance of 10−5 Å. The coupling parameters of temperature and pressure were set to 1.0 ps. For the sampling parameters, the coordinates of total complex were saved with a time interval of 0.1 ps.

### Animals

All animal studies were conducted in compliance with the Guide of Chinese Academy of Medical Sciences and approved by the Animal Ethics Committees of the West China Hospital of Sichuan University. The balb/c mice at an age of four to six weeks with body weights ranging from 18 to 22 g were obtained from the Beijing Huafukang Laboratory Animal Limited Company. The mice were maintained in stable environment temperature at 20-25 °C and relative humidity about 55-60%.The mice were maintained under the guidelines of the National Science Council of the People's Republic of China.

### Immunization procedure

Mice were used at 8 weeks to begin the experiments. In animal study of the new vaccines, four and five groups of mice were included in the two experiments. Model group received subcutaneously immunization with saline, challenged and exposure to OVA. Immunization groups were injected respectively and subcutaneously with vaccine which had equal amount to 100ug IL-13, subjected to intraperitoneal sensitization and challenged with ovalbumin (OVA) (Sigma-Aldrich).Blood samples were collected on week 1,3,5,7,9,11 and the endpoint of the whole experiment. Moreover, thirty-six to seventy-two hours after OVA exposure or no exposure, all mice should be sacrificed and their blood samples need be collected to test antibodies and cytokines. Bronchoalveolar Lavage Fluids (BALF) of the mice were collected with three repeated washes using 1ml pre-cold(4°C) PBS or saline. The all samples of BALF were centrifuged at 4°C for 10 min (1,200 rpm). Supernatants of BALF were collected and stored at −80°C for the following measurements by a series of ELISA.

### Antibodies and cytokines measured by enzyme-linked immuno-sorbent assay

As described before, blood samples were collected on week 1,3,5,7,9,11 and the endpoint of the whole experiment, stored at −80°C. The serum level of IL-13-specific IgG, IgG1, IgG2a, OVA-specific IgG and OVA-specific IgE were assayed by enzyme-linked immune-sorbent assay(ELISA). Microplates were coated with either mouse IL-13(1ug/ml) or OVA(1ug/ ml) at 4 °C overnight, closed with skimmed milk, incubated serum samples diluting 2-fold serially, incubated with alkaline phosphatase-conjugated goat anti-mouse IgG or alkaline phosphatase-conjugated goat anti-mouse IgE, and last added stop buffer following substrate to end the reaction. The concentration of IL-4,IL-13,IFN-γ and total IgE were detected using commercial ELISA kits. The IL-4 and IL-13 enzyme-linked immunosorbent assay (ELISA) kit were purchased from R&D Systems; the IFN-γ ELISA kit were from Invitrogen; the total IgE ELISA kit from MD Biosciences.

### Histologic assessment

After sacrificing the mice, their lungs were collected and fixed in 4% formalin. The lung tissues were embedded in paraffin, sectioned(5-μm thick) and placed on glass slides for the histological examination. Sections of lungs were heated on 1-2h at 65°C,stained with hematoxylin and eosin (H&E) or alcian blue-periodic acid schiff (PAS) to detect inflammation, mucus production and examined goblet cells proliferation using an Olympus light microscope equipped with a camera. Mucin-secreted cells called goblet cells were stained by Alcain blue-PAS and measured by the percentage of the staining positive cells in the total airway epithelia of medium-sized airway.

After sacrificing the mice, tissues were collected and fixed in 4% formalin. All tissues were embedded in paraffin, sectioned (5-μm thick) and placed on glass slides for the histological examination. Sections of tissues were heated on 1-2h at 65°C,stained with hematoxylin and eosin (H&E) detect whether vaccination had effects on other tissues or not by an Olympus light microscope equipped with a camera. At the meantime, weight of mice was measured during and at the endpoint the experiment. At the end point of experiment, weight of mice and lung in mice was compared to analyze the effects of vaccination.

### Image processing software

Images were built using Adobe Photoshop CS6 (ver: 13.0, Adobe Systems Software Ireland Ltd, CA, USA) and formatted using Adobe Illustrator CS6 (ver: 16.0, Adobe Systems Software Ireland Ltd, CA, USA).

### Statistical analysis

All values were reported as the means ± SEM and the significant differences between groups were analyzed by one-way ANOVA followed by Newman-Keuls multiple comparison test. Statistical signifi¬cance was set at *P* < 0.05.

## CONCLUSIONS

In conclusion, we designed and synthesized IL-13 peptide vaccine candidates by integrative in silico and experimental screening. These vaccines comprised a T-cell helper epitope came from different sources and a non-immunogenic pathogenic epitope. Immunological evaluations showed that the vaccines with N-terminal fragment of IL-13 and Ditox epitope elicited significant immune response, down-regulated cytokines in BALF or serum, suppressed inflammation/AHR in mouse asthma model and used the lysosome to cross-presenting MHC II to activate CD4+ T cell responses. These totally synthetic IL-13 peptide−T-cell helper epitope conjugate suggests a novel peptide vaccine discovery strategy, with simple and well-define structure that should deserve further studies. Our approach offers new possibilities to discovery therapeutic peptide vaccine candidates for chronic non-infectious diseases, with highly consolidated in silico and animal disease models for fast iterative screening.

## SUPPLEMENTARY MATERIALS FIGURES AND TABLES




