# Supplementary material for: Interleukin-13 peptide vaccine induces protective humoral immunity in murine asthma models
Source: Oncotarget. 2017 Aug 4;9(6):6678–90. doi: 10.18632/oncotarget.19950 (PMC5805505; doi:10.18632/oncotarget.19950)
Supplement: Supplementary file 1 [file oncotarget-09-6678-s001.pdf]

Interleukin-13 peptide vaccine induces protective humoral immunity in murine asthma models

SUPPLEMENTARY MATERIALS

Table of Contents

1. Regents and materials

2. General methods for peptide synthesis

3. Tables and figures

4. HPLC chromatograms and mass spectrums

1. Regents and materials

The synthetic peptide vaccines were synthesized by standard solid-phase peptide synthesis (SPPS) protocols on a CSBio 136XT peptide synthesis instrument. The peptides were purified by high-performance liquid chromatography (HPLC) to 95% purity. The molecular weights were confirmed by mass spectrometry. Female BALB/c mice (6-8 wk old) were purchased from Beijing Wei Tong Li Hua Biological Technology Co. Ltd. (Beijing, China). And all of the experimental protocols were

approved by the Institutional Animal Care and Use Committee of Sichuan University (Chengdu, China). Mice were used at 8 weeks to begin the experiments.

2. General methods for peptide synthesis

Fmoc-solid phase peptide synthesis was assisted by a CSBio 136XT peptide synthesizer and/or magnetic shaking at 0.1 mmol scale on Rink amide MBHA resin. Resin loading for the linear peptides was 0.39 mmol/g, and all resins were previously swollen in DMF before use. The pre-activated amino acids (5.0 equiv per mol amino group) were prepared by dissolving them in a HBTU/DMF (4.0 equiv) and DIPEA (7.0 equiv) solution were added and stirred for 25 min. After the amino acids were coupled completely, the Fmoc group were removed by piperidine in DMF (20% v/v) and DMF washing of the resin twice. The coupling of Fmoc-His(trt)-OH and Fmoc-Arg(pbf)-OH was caried out at for a longer time for higher coupling efficiency. After the synthesis was completed and the terminal Fmoc group removed, the resin was washed with DMF, MeOH and DCM, respectively, and dried in vacuo. The crude peptides were cleaved from the resin by using incubation with TFA/TIS/water (95:2.5:2.5%). All cleaved peptides were precipitated in diethyl ether, filtered and washed with diethyl ether thoroughly. The precipitated peptides were dissolved in acetonitrile/water (1:1) that contained 0.1% TFA for further purified using RP-HPLC.

Preparative RP-HPLC separation of crude peptides was performed on a Waters-600 instrument with solution A (acetonitrile with 0.1% trifluoroacetic acid) and solution B (pure water with 0.1% trifluoroacetic acid). The absorption was detected by a

Waters-486 UV detector at 215nm. The semi-preparative C18 column (YMC, 10μm, 20 × 250 mm) with a flow rate of 10.0 ml/min and a preparative C18 column (Novasep, 10μm, 50 × 250 mm) with a flow rate of 50.0 ml/min were used for separation crude peptides. The pure products were characterized by ESI-MS, and then lyophilized and stored at -20°C. Before use, all peptides were re-analyzed by analytical RP-HPLC on Shimadzu LC-20A instrument with an analytic C18 column (Intersil, 5μm, 4.6×250 mm) with a flow rate of 1.0 ml/min.

3. Tables and figures

Supplementary Table 1: The HLA_DR alleles and the coverage in Chinese population.

Alleles	Percentage of individuals that have the allele	Coverage
DRB1*09:01	25.2	0.135
DRB1*07:01	23.3	0.124
DRB1*15:01	22.3	0.119
DRB1*15:02	16.5	0.086
DRB1*11:01	11.7	0.06
DRB1*12:02	11.7	0.06
DRB1*04:05	9.7	0.05
DRB1*04:06	8.7	0.045
DRB1*16:02	8.7	0.045
DRB1*14:01	7.8	0.04
DRB1*08:03	6.8	0.035
DRB1*03:01	5.8	0.03
DRB1*01:01	4.9	0.025
DRB1*12:01	4.9	0.025
DRB1*04:01	3.9	0.02
DRB1*10:01	2.9	0.015
DRB1*13:01	2.9	0.015
DRB1*04:03	1.9	0.01
DRB1*08:02	1.9	0.01
DRB1*14:03	1.9	0.01

For Supplementtray Table 2 see in Supplementary Files

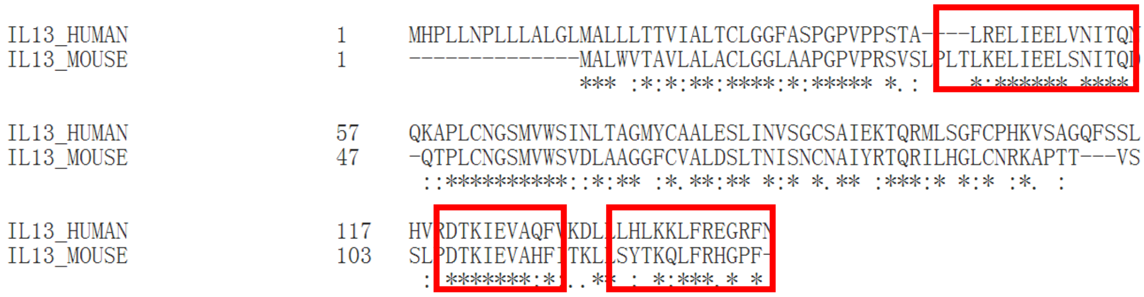

Supplementary Figure 1: The sequence alignment of IL13_Human and IL13_Mouse.

4. HPLC chromatograms and mass spectrums

V1: AYNFVESIINLFQVVHNSYN-DTKIEVAHF

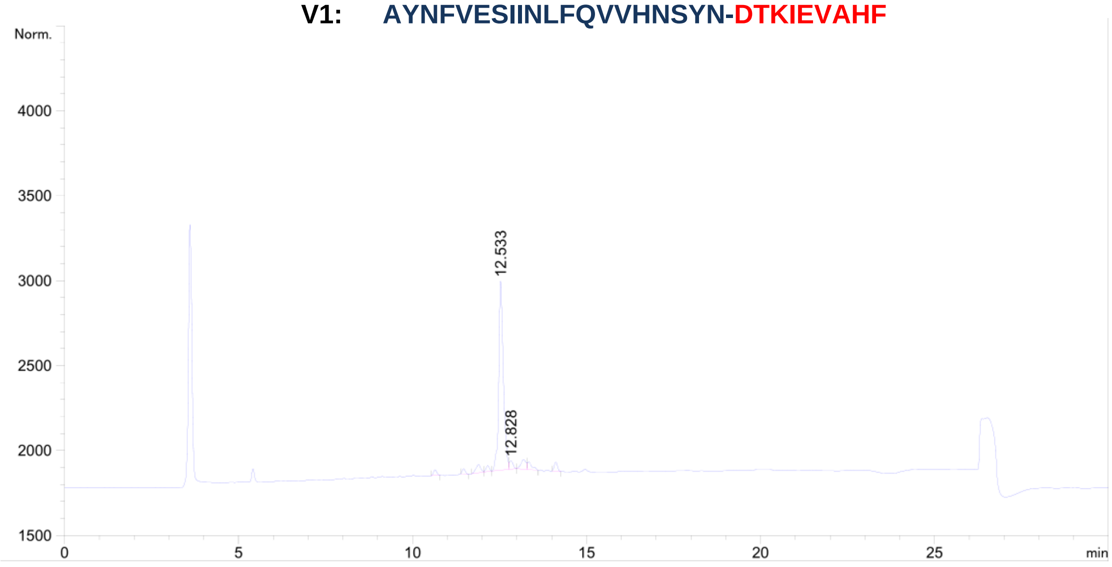

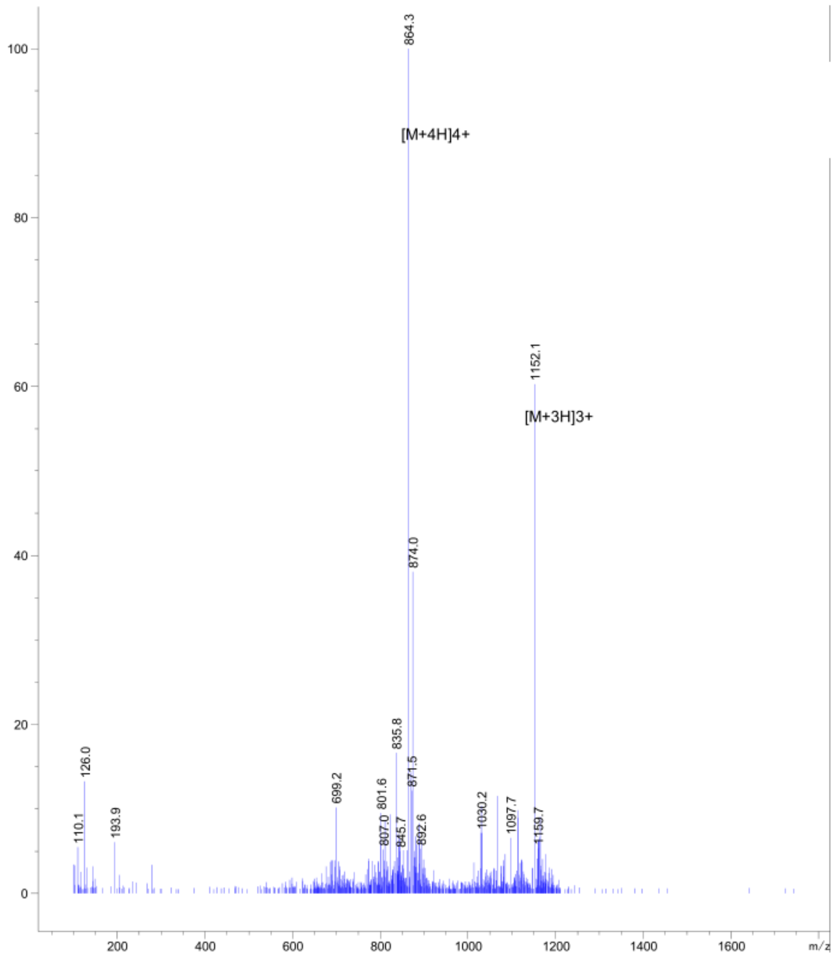

V2: AYNFVESIINLFQVVHNSYN-SYTKQLFRHGPF

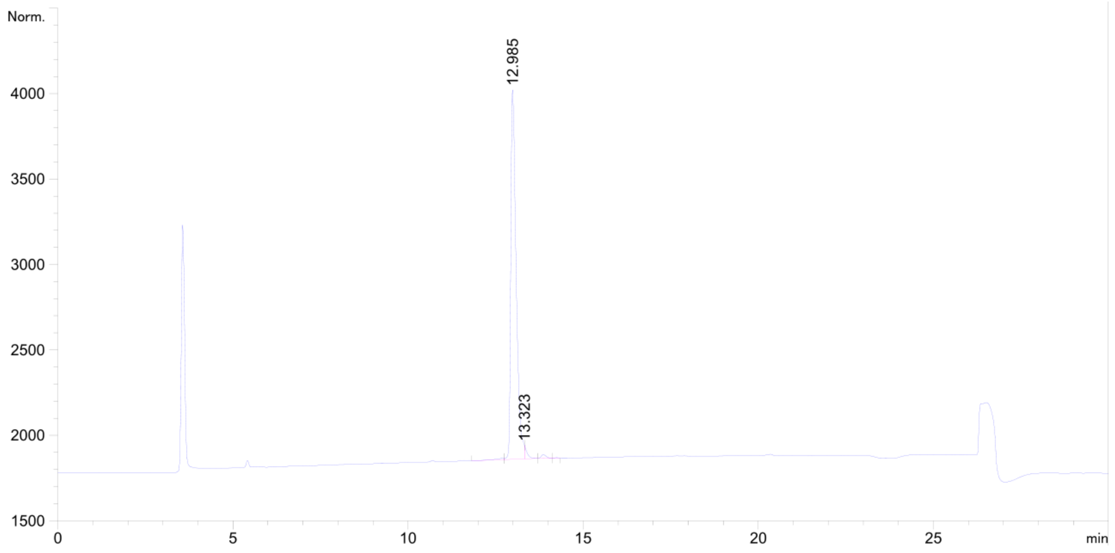

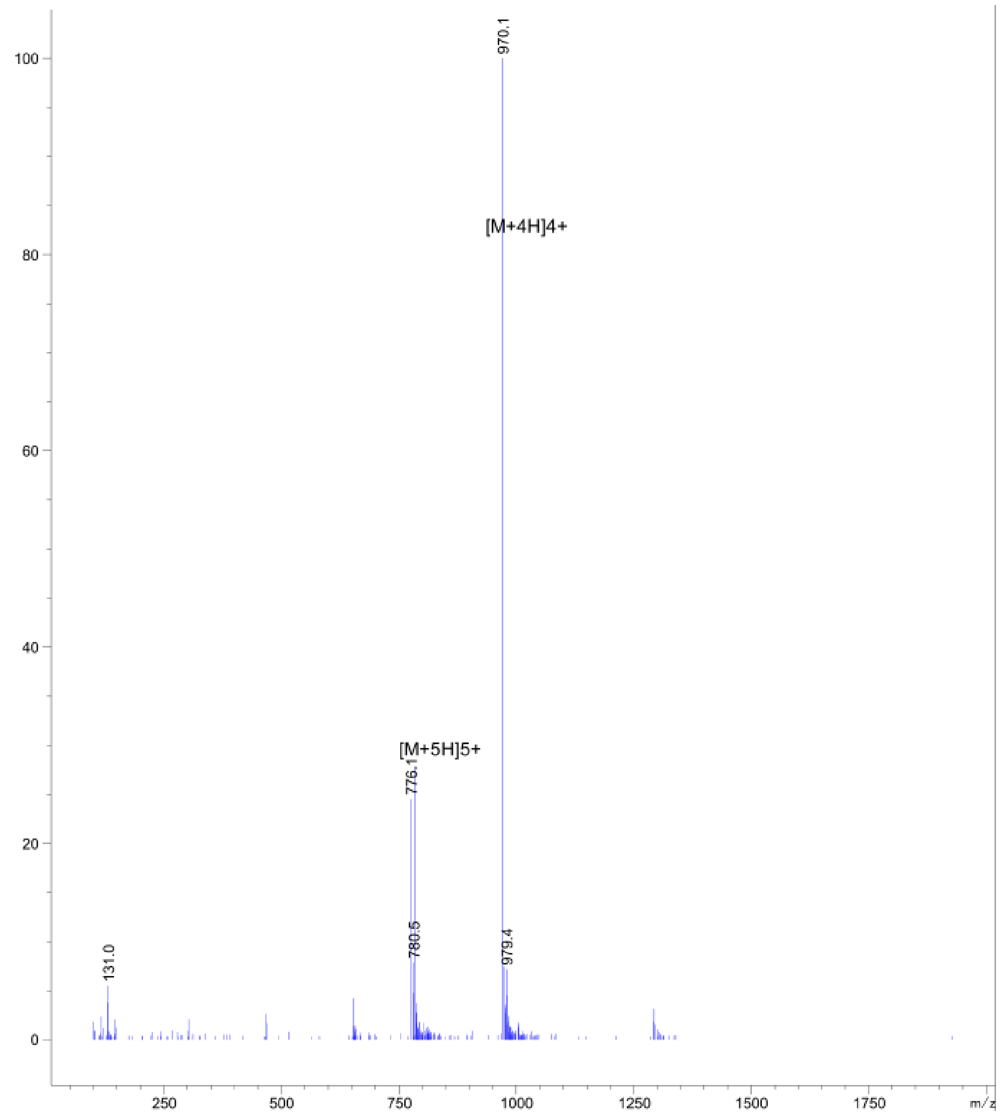

V3: AYNFVESIINLFQVVHNSYN-LTLKELIEELSNITQ

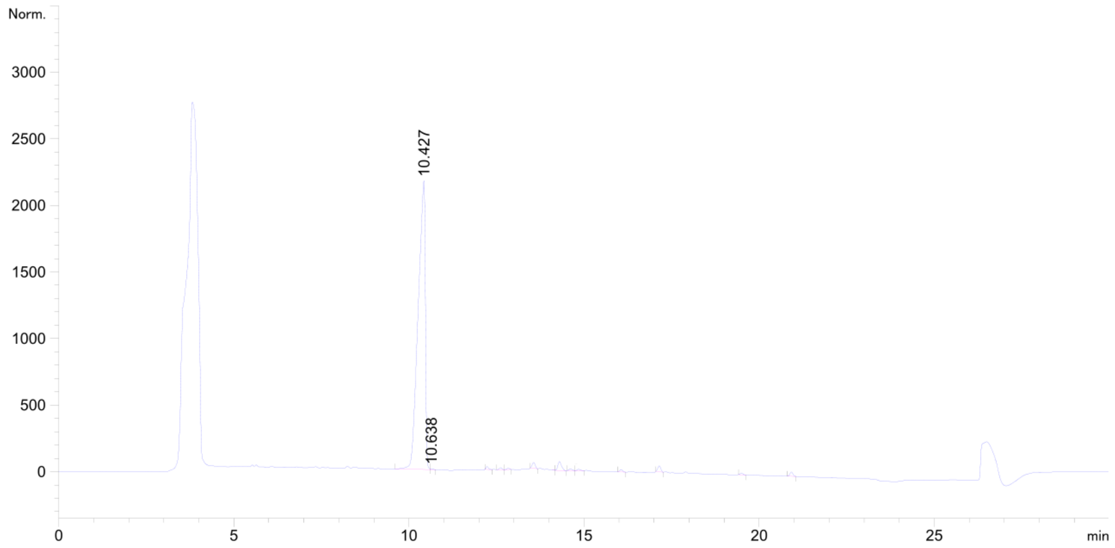

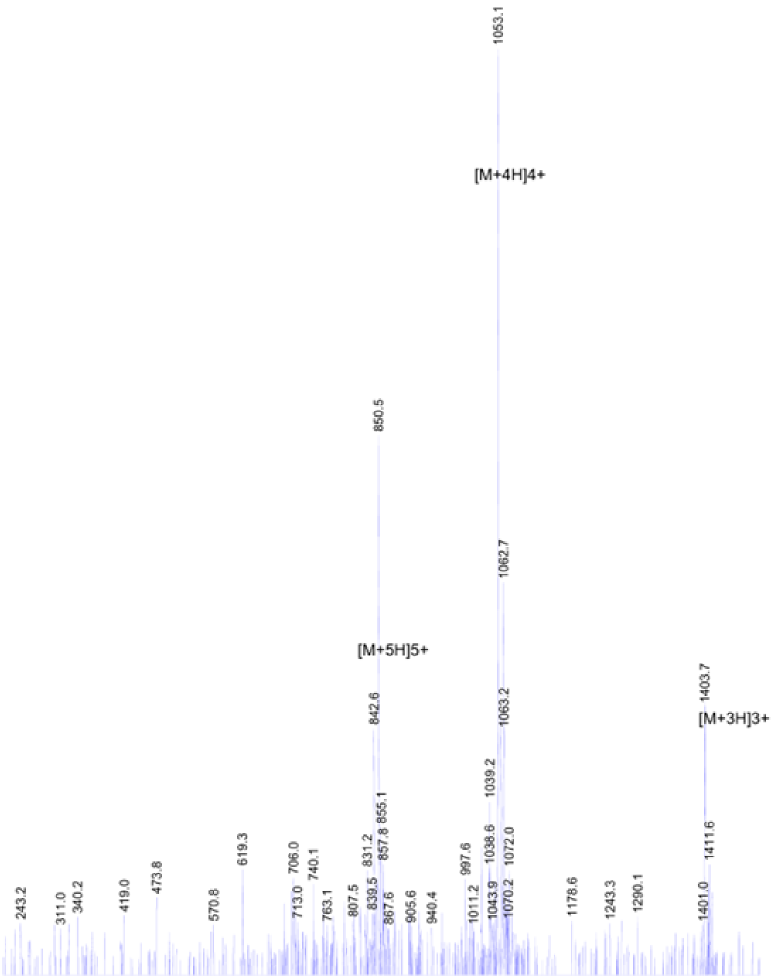

V4: QYIKANSKFIGITE-Ahx-LTLKELIEELSNITQ

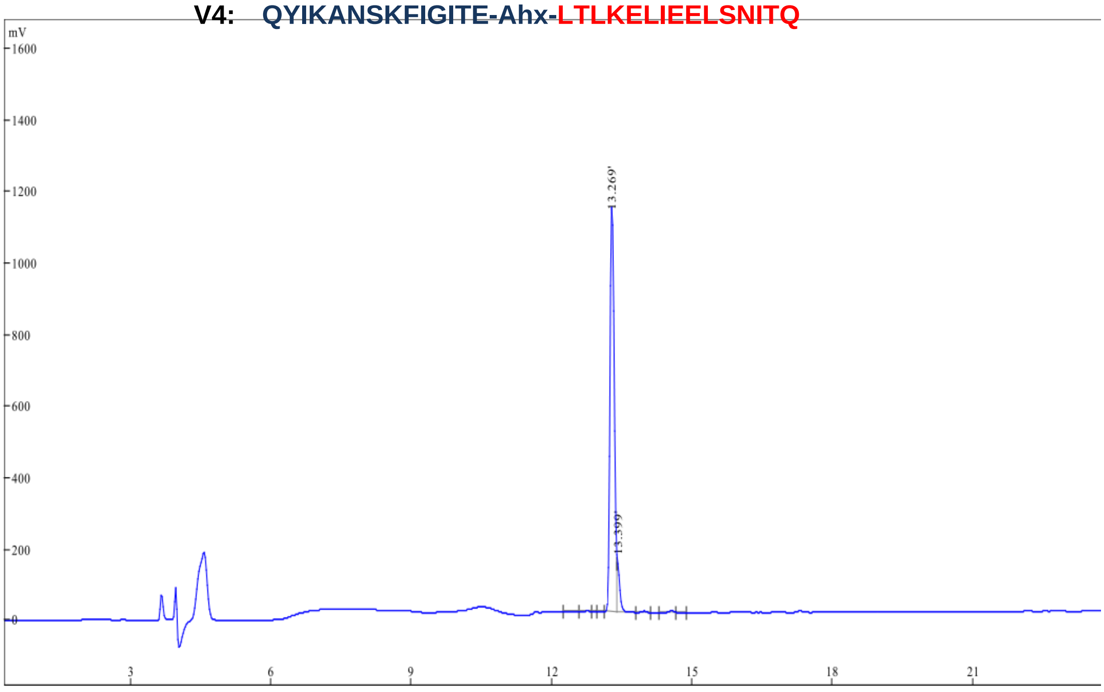

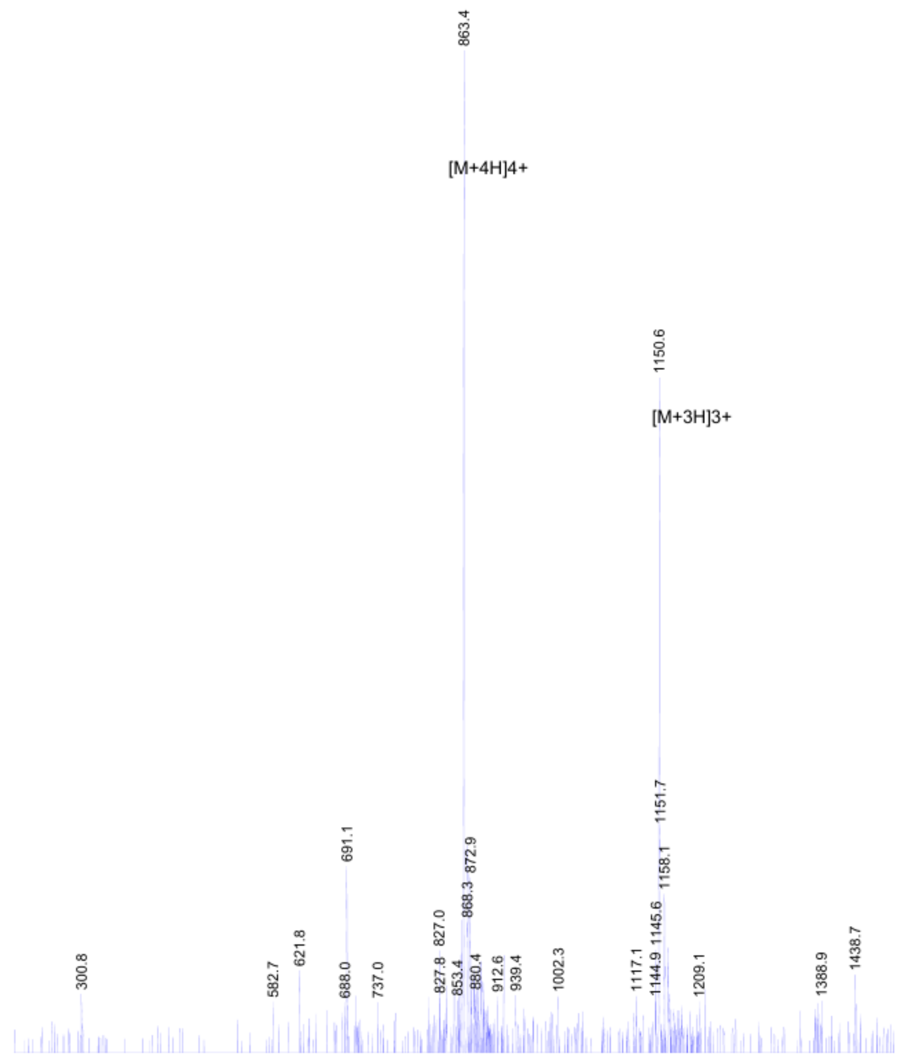

V5: FNNFTVSFWLRVPKVSASHLE-Ahx-LTLKELIEELSNITQ

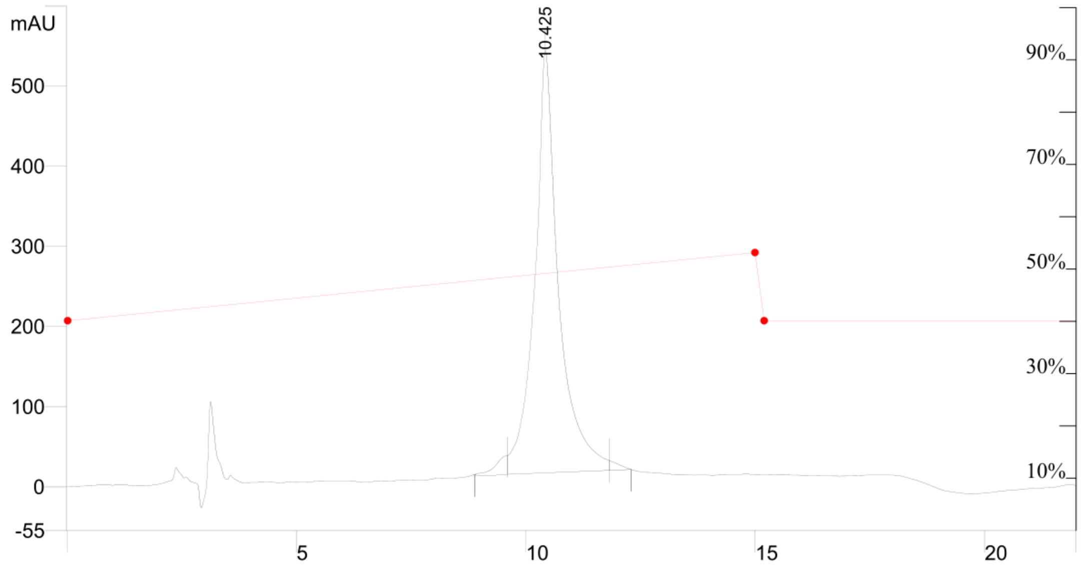

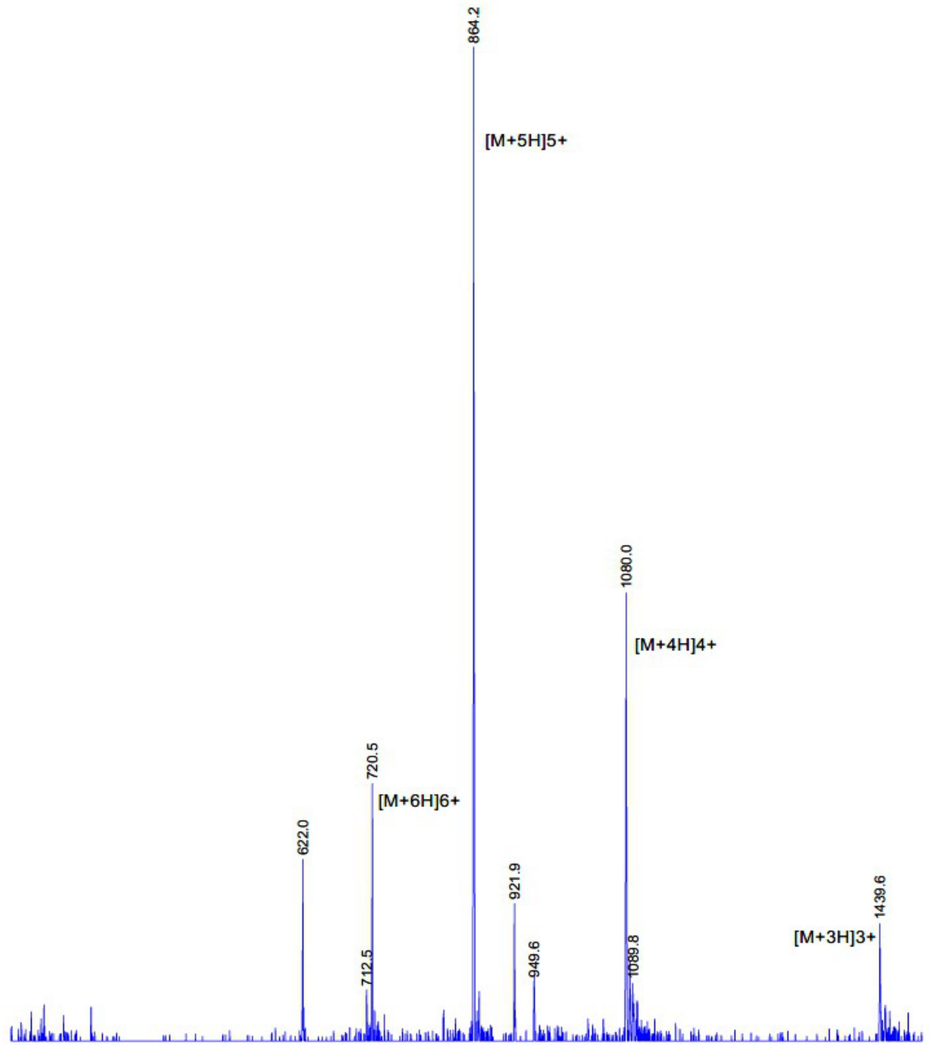

V6: aK-Cha-VAAWTLKAa-Ahx-LTLKELIEELSNITQ

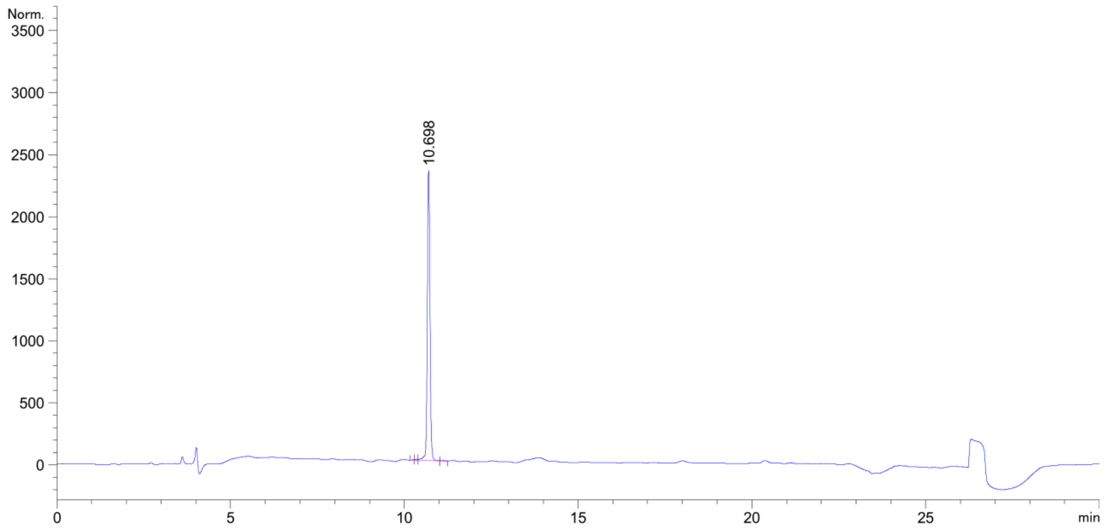

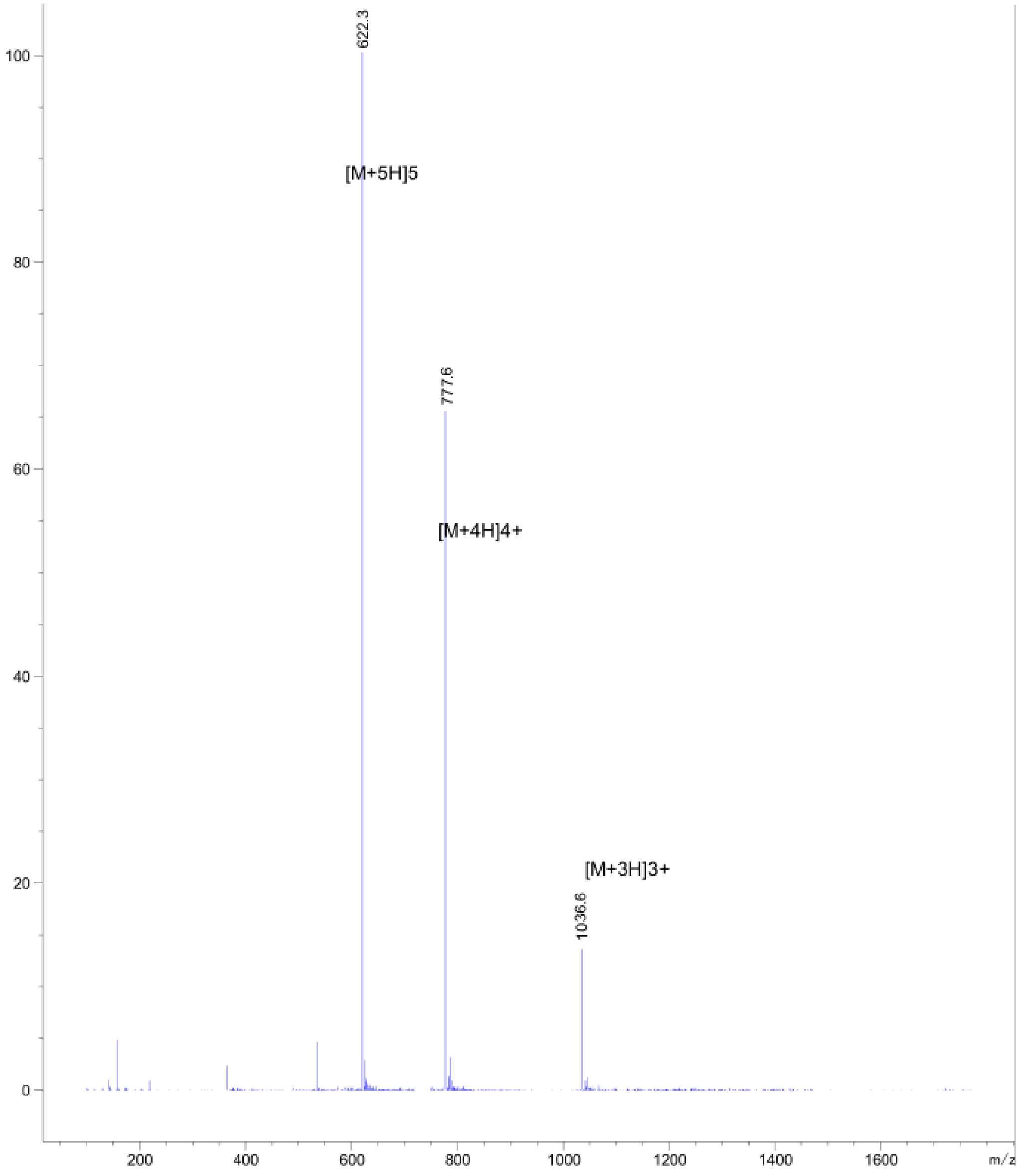

622.3
[M+5H]5
777.6
[M+4H]4+
[M+3H]3+
1036.6
100
80
60
40
20
0
200
400
600
800
1000
1200
1400
1600
m/z
