# Supplementary material for: Interleukin-13 peptide vaccine induces protective humoral immunity in murine asthma models
Source: Oncotarget. 2017 Aug 4;9(6):6678–90. doi: 10.18632/oncotarget.19950 (PMC5805505; doi:10.18632/oncotarget.19950)
Supplement: Supplementary file 2 [file oncotarget-09-6678-s002.docx]

**Supplementary Table 2: Predicted IL-13 epitopes for Chinese population.**

| allele | peptide | percentile_rank |
| --- | --- | --- |
| HLA-DRB1*11:01 | LHLKKLFREGRF | 0.56 |
| HLA-DRB1*11:01 | LHLKKLFREGRFN | 0.56 |
| HLA-DRB1*07:01 | DTKIEVAQF | 0.56 |
| HLA-DRB1*11:01 | ALRELIEELVNITQ | 0.57 |
| HLA-DRB1*11:01 | LRELIEELVNITQ | 0.57 |
| HLA-DRB1*11:01 | LRELIEELVNITQN | 0.57 |
| HLA-DRB1*07:01 | LLLTTVIAL | 0.57 |
| HLA-DRB1*01:01 | ALGLMALLL | 0.57 |
| HLA-DRB1*07:01 | LGLMALLLTTVIALT | 0.57 |
| HLA-DRB1*07:01 | GLMALLLTTVIALTC | 0.57 |
| HLA-DRB1*07:01 | LMALLLTTVIALTCL | 0.57 |
| HLA-DRB1*07:01 | ALGLMALLLTTVIAL | 1.36 |
| HLA-DRB1*01:01 | NPLLLALGLMALLLT | 1.36 |
| HLA-DRB1*01:01 | LLALGLMALLLTTVI | 1.53 |
| HLA-DRB1*09:01 | AGMYCAALESLINVS | 1.53 |
| HLA-DRB1*01:01 | LLNPLLLALGLMALL | 1.53 |
| HLA-DRB1*01:01 | PLLLALGLMALLLTT | 1.53 |
| HLA-DRB1*01:01 | LLLALGLMALLLTTV | 1.53 |
| HLA-DRB1*09:01 | TAGMYCAALESLINV | 1.53 |
| HLA-DRB1*01:01 | PLLNPLLLALGLMAL | 1.53 |
| HLA-DRB1*01:01 | LALGLMALLLTTVIA | 1.53 |
| HLA-DRB1*09:01 | LTAGMYCAALESLIN | 1.82 |
| HLA-DRB1*07:01 | ALLLTTVIALTCLGG | 1.82 |
| HLA-DRB1*09:01 | GMYCAALESLINVSG | 1.82 |
| HLA-DRB1*07:01 | LLLTTVIALTCLGGF | 1.82 |
| HLA-DRB1*01:01 | ALGLMALLLTTVIAL | 1.82 |
| HLA-DRB1*01:01 | LGLMALLLTTVIALT | 2.01 |
| HLA-DRB1*11:01 | NPLLLALGLMALLLT | 2.01 |
| HLA-DRB1*11:01 | PLLLALGLMALLLTT | 2.21 |
| HLA-DRB1*11:01 | LLLALGLMALLLTTV | 2.21 |
| HLA-DRB1*11:01 | LLALGLMALLLTTVI | 2.21 |
| HLA-DRB1*11:01 | LALGLMALLLTTVIA | 2.21 |
| HLA-DRB1*11:01 | ALGLMALLLTTVIAL | 2.28 |
| HLA-DRB1*11:01 | LGLMALLLTTVIALT | 2.29 |
| HLA-DRB1*15:01 | NPLLLALGLMALLLT | 2.32 |
| HLA-DRB1*15:01 | LLALGLMALLLTTVI | 2.44 |
| HLA-DRB1*11:01 | HPLLNPLLLALGLMA | 2.44 |
| HLA-DRB1*11:01 | PLLNPLLLALGLMAL | 2.46 |
| HLA-DRB1*11:01 | LLNPLLLALGLMALL | 2.54 |
| HLA-DRB1*11:01 | LNPLLLALGLMALLL | 2.61 |
| HLA-DRB1*15:01 | LNPLLLALGLMALLL | 2.64 |
| HLA-DRB1*09:01 | NLTAGMYCAALESLI | 2.65 |
| HLA-DRB1*15:01 | LALGLMALLLTTVIA | 2.65 |
| HLA-DRB1*15:01 | LLLALGLMALLLTTV | 2.65 |
| HLA-DRB1*11:01 | LTTVIALTCLGGFAS | 2.68 |
| HLA-DRB1*15:01 | ALGLMALLLTTVIAL | 2.7 |
| HLA-DRB1*15:01 | LGLMALLLTTVIALT | 2.73 |
| HLA-DRB1*15:01 | QFVKDLLLHLKKLFR | 2.74 |
| HLA-DRB1*15:01 | FVKDLLLHLKKLFRE | 2.77 |
| HLA-DRB1*11:01 | CNGSMVWSINLTAGM | 2.77 |
| HLA-DRB1*11:01 | NGSMVWSINLTAGMY | 2.9 |
| HLA-DRB1*11:01 | GSMVWSINLTAGMYC | 2.9 |
| HLA-DRB1*11:01 | ALLLTTVIALTCLGG | 2.9 |
| HLA-DRB1*11:01 | AQFVKDLLLHLKKLF | 2.9 |
| HLA-DRB1*15:01 | PLLNPLLLALGLMAL | 2.9 |
| HLA-DRB1*15:01 | LLNPLLLALGLMALL | 2.9 |
| HLA-DRB1*11:01 | TTVIALTCLGGFASP | 2.95 |
| HLA-DRB1*11:01 | VSGCSAIEKTQRMLS | 3 |
| HLA-DRB1*11:01 | SGCSAIEKTQRMLSG | 3 |
| HLA-DRB1*11:01 | GCSAIEKTQRMLSGF | 3 |
| HLA-DRB1*11:01 | CSAIEKTQRMLSGFC | 3 |
| HLA-DRB1*11:01 | SAIEKTQRMLSGFCP | 3.11 |
| HLA-DRB1*11:01 | AIEKTQRMLSGFCPH | 3.12 |
| HLA-DRB1*11:01 | IEKTQRMLSGFCPHK | 3.13 |
| HLA-DRB1*07:01 | KIEVAQFVKDLLLHL | 3.13 |
| HLA-DRB1*15:01 | PLLLALGLMALLLTT | 3.49 |
| HLA-DRB1*11:01 | SMVWSINLTAGMYCA | 3.57 |
| HLA-DRB1*11:01 | GLMALLLTTVIALTC | 3.74 |
| HLA-DRB1*11:01 | LMALLLTTVIALTCL | 3.75 |
| HLA-DRB1*11:01 | MALLLTTVIALTCLG | 3.92 |
| HLA-DRB1*11:01 | MVWSINLTAGMYCAA | 3.92 |
| HLA-DRB1*09:01 | MYCAALESLINVSGC | 3.92 |
| HLA-DRB1*09:01 | GSMVWSINLTAGMYC | 3.92 |
| HLA-DRB1*01:01 | GLMALLLTTVIALTC | 3.92 |
| HLA-DRB1*11:01 | LLLTTVIALTCLGGF | 3.93 |
| HLA-DRB1*09:01 | NGSMVWSINLTAGMY | 4.01 |
| HLA-DRB1*11:01 | TVIALTCLGGFASPG | 4.02 |
| HLA-DRB1*15:01 | HPLLNPLLLALGLMA | 4.22 |
| HLA-DRB1*11:01 | VIALTCLGGFASPGP | 4.23 |
| HLA-DRB1*11:01 | KIEVAQFVKDLLLHL | 4.26 |
| HLA-DRB1*11:01 | IEVAQFVKDLLLHLK | 4.32 |
| HLA-DRB1*11:01 | EVAQFVKDLLLHLKK | 4.33 |
| HLA-DRB1*11:01 | VAQFVKDLLLHLKKL | 4.62 |
| HLA-DRB1*15:01 | MHPLLNPLLLALGLM | 4.7 |
| HLA-DRB1*07:01 | IEVAQFVKDLLLHLK | 4.7 |
| HLA-DRB1*09:01 | CNGSMVWSINLTAGM | 4.7 |
| HLA-DRB1*11:01 | LCNGSMVWSINLTAG | 4.7 |
| HLA-DRB1*07:01 | CPHKVSAGQFSSLHV | 4.7 |
| HLA-DRB1*15:01 | GLMALLLTTVIALTC | 4.7 |
| HLA-DRB1*07:01 | SAGQFSSLHVRDTKI | 4.7 |
| HLA-DRB1*09:01 | QRMLSGFCPHKVSAG | 4.73 |
| HLA-DRB1*11:01 | RELIEELVNITQNQK | 4.75 |
| HLA-DRB1*11:01 | ELIEELVNITQNQKA | 4.86 |
| HLA-DRB1*11:01 | LIEELVNITQNQKAP | 4.93 |
